# Supplementary material for: Differential effects of physical activity on cognitive and motor performance in obese young adults
Source: PeerJ. 2025 Dec 9;13:e20481. doi: 10.7717/peerj.20481 (PMC12700113; doi:10.7717/peerj.20481)
Supplement: Supplemental Information 1 [file peerj-13-20481-s001.doc]

STROBE Statement—Checklist of items that should be included in reports of ***cross-sectional studies***

|  | Item No | Recommendation |
| --- | --- | --- |
| **Title and abstract** | 1 | (*a*) Indicate the study’s design with a commonly used term in the title or the abstract  Cross-sectional study as stated in the Abstract on page 2 and Materials and methods on page 4. |
|  |  | (*b*) Provide in the abstract an informative and balanced summary of what was done and what was found  Provided in Abstract on page 2. |
| Introduction | | |
| Background/rationale | 2 | Explain the scientific background and rationale for the investigation being reported  Included in the Introduction on pages 3 and 4. |
| Objectives | 3 | State specific objectives, including any prespecified hypotheses  Included in the Introduction on page 4. |
| Methods | | |
| Study design | 4 | Present key elements of study design early in the paper  Included in the Materials and methods on page 4 |
| Setting | 5 | Describe the setting, locations, and relevant dates, including periods of recruitment, exposure, follow-up, and data collection  Included in the Methods on pages 4 |
| Participants | 6 | (*a*) Give the eligibility criteria, and the sources and methods of selection of participants  Included in Study participants on page 4-5 |
| Variables | 7 | Clearly define all outcomes, exposures, predictors, potential confounders, and effect modifiers. Give diagnostic criteria, if applicable  Included in Procedure and Cognitive assessment on page 5-7 |
| Data sources/ measurement | 8* | For each variable of interest, give sources of data and details of methods of assessment (measurement). Describe comparability of assessment methods if there is more than one group  Detailed in Cognitive assessment and Gait speed assessment on page 6-7 |
| Bias | 9 | Describe any efforts to address potential sources of bias  Matching for sex and BMI described on page 5; limitations mention self-report bias on page 10 |
| Study size | 10 | Explain how the study size was arrived at  G*Power calculation described in Study participants on page 4-5 |
| Quantitative variables | 11 | Explain how quantitative variables were handled in the analyses. If applicable, describe which groupings were chosen and why  Grouped based on MET-min/week; cutoffs specified include in Procedure on page 5 |
| Statistical methods | 12 | (*a*) Describe all statistical methods, including those used to control for confounding  Included in the Statistical analysis on page 7 |
|  |  | (*b*) Describe any methods used to examine subgroups and interactions  Not reported |
|  |  | (*c*) Explain how missing data were addressed  Not reported |
|  |  | (*d*) If applicable, describe analytical methods taking account of sampling strategy  Not applicable |
|  |  | (*e*) Describe any sensitivity analyses  Not applicable |
| Results | | |
| Participants | 13* | (a) Report numbers of individuals at each stage of study—eg numbers potentially eligible, examined for eligibility, confirmed eligible, included in the study, completing follow-up, and analysed  Included in the Procedure on page 5 |
|  |  | (b) Give reasons for non-participation at each stage  Not reported |
|  |  | (c) Consider use of a flow diagram  Figure 1 provides flowchart |
| Descriptive data | 14* | (a) Give characteristics of study participants (eg demographic, clinical, social) and information on exposures and potential confounders  Table 1 and text in Results section |
|  |  | (b) Indicate number of participants with missing data for each variable of interest  Not reported |
| Outcome data | 15* | Report numbers of outcome events or summary measures  Results Tables 1 & 2 |
| Main results | 16 | (*a*) Give unadjusted estimates and, if applicable, confounder-adjusted estimates and their precision (eg, 95% confidence interval). Make clear which confounders were adjusted for and why they were included  Unadjusted t-test results provided |
|  |  | (*b*) Report category boundaries when continuous variables were categorized  600 MET-min/week cut-off used to define groups included in Procedure page 5 |
|  |  | (*c*) If relevant, consider translating estimates of relative risk into absolute risk for a meaningful time period  Not applicable |
| Other analyses | 17 | Report other analyses done—eg analyses of subgroups and interactions, and sensitivity analyses  Not applicable. |
| Discussion | | |
| Key results | 18 | Summarise key results with reference to study objectives  Included in Discussion on page 8-10 |
| Limitations | 19 | Discuss limitations of the study, taking into account sources of potential bias or imprecision. Discuss both direction and magnitude of any potential bias  Included in Discussion on page 8-10 |
| Interpretation | 20 | Give a cautious overall interpretation of results considering objectives, limitations, multiplicity of analyses, results from similar studies, and other relevant evidence  Final paragraphs of Discussion on page 10 |
| Generalisability | 21 | Discuss the generalisability (external validity) of the study results  Briefly mentioned in limitations on page 9-10 |
| Other information | | |
| Funding | 22 | Give the source of funding and the role of the funders for the present study and, if applicable, for the original study on which the present article is based  Acknowledgement section on page 11 |
